# Continuous Ethanol Production with a Membrane Bioreactor at High Acetic Acid Concentrations

**DOI:** 10.3390/membranes4030372

**Published:** 2014-07-15

**Authors:** Päivi Ylitervo, Carl Johan Franzén, Mohammad J. Taherzadeh

**Affiliations:** 1 Swedish Centre for Resource Recovery, University of Borås, 501 90 Borås, Sweden; E-Mail: mohammad.taherzadeh@hb.se; 2 Chemical and Biological Engineering—Industrial Biotechnology, Chalmers University of Technology, 412 96 Göteborg, Sweden; E-Mail: franzen@chalmers.se

**Keywords:** acetic acid, membrane bioreactor, bioethanol, cell retention, yeast

## Abstract

The release of inhibitory concentrations of acetic acid from lignocellulosic raw materials during hydrolysis is one of the main concerns for 2nd generation ethanol production. The undissociated form of acetic acid can enter the cell by diffusion through the plasma membrane and trigger several toxic effects, such as uncoupling and lowered intracellular pH. The effect of acetic acid on the ethanol production was investigated in continuous cultivations by adding medium containing 2.5 to 20.0 g·L^−1^ acetic acid at pH 5.0, at a dilution rate of 0.5 h^−1^. The cultivations were performed at both high (~25 g·L^−1^) and very high (100–200 g·L^−1^) yeast concentration by retaining the yeast cells inside the reactor by a cross-flow membrane in a membrane bioreactor. The yeast was able to steadily produce ethanol from 25 g·L^−1^ sucrose, at volumetric rates of 5–6 g·L^−1^·h^−1^ at acetic acid concentrations up to 15.0 g·L^−1^. However, the yeast continued to produce ethanol also at a concentration of 20 g·L^−1^ acetic acid but at a declining rate. The study thereby demonstrates the great potential of the membrane bioreactor for improving the robustness of the ethanol production based on lignocellulosic raw materials.

## 1. Introduction

Today, the transportation sector consumes more than half of the petroleum used in the world, and the demand for fuel is expected to increase in the future as vehicle traffic becomes more abundant in especially Asia [1]. The utilization of fossil fuel has large negative impact on the environment, and it is of great importance to increase the production of renewable fuels such as ethanol. First generation ethanol production from starch and sugar-rich feedstock is already a mature process [2]. Unfortunately, these feedstocks cannot meet the increased demand for fuel, and concerns have been raised about the use of food crops for fuel production [3]. An alternative is to utilize lignocellulosic materials as raw material in the fermentation process for ethanol production, since lignocellulosic materials, such as residues from agriculture and forestry, are renewable, abundant and still relatively cheap [2].

Regardless of the feedstock, the final ethanol price must be competitive with the gasoline prize. Gasoline benefits from that the technology is already well developed because of over a century of learning and optimization. Profit margins for ethanol production processes in contrast are usually low, and the return on capital is unsure [1].

Continuous fermentation processes offer some beneficial traits that may lead to reduced process costs during ethanol production [1]. An important measurement for evaluation of the performance of a fermentation system is its productivity, *i.e.*, the amount of produced product per unit of time and reactor volume. The productivity depends on several factors, such as the concentrations of substrates and cells, the specific product formation rate, and the dilution rate. Generally, a continuous process can provide a higher volumetric productivity compared to a repeated batch process. Additionally, an almost complete utilization of the substrate is desired, in order to avoid unproductive loss of substrate [1,4]. In Brazil, it is today most common to perform ethanolic fermentation in fed-batch mode, in order to avoid high substrate concentrations which result in inhibition of metabolic processes in *S. cerevisiae*. However, about 20%–30% of the distilleries use continuous fermentation processes. One problem with continuous processes is that they are prone to bacterial contamination. In batch and fed-batch cultures, bacterial contamination can be reduced even when recycling the cells, by treating the cells with dilute sulfuric acid to kill contaminating bacteria [4].

Utilizing lignocellulose for ethanol production is complicated due to the recalcitrant nature of the raw materials. Therefore, harsh treatments, e.g., with chemicals and/or high temperatures, are needed in order to degrade lignocellulose to fermentable sugars [5]. Dilute acid hydrolysis is a common and rather simple method to hydrolyze the lignocellulose to sugars. Unfortunately, several compounds that inhibit fermentation are also formed in the process, such as aliphatic acids, furan aldehydes, and phenolic compounds.

Acetic acid is an important inhibitor in lignocellulosic hydrolyzates. Acetic acid is released during solubilization and hydrolysis of hemicellulose [6]. The concentration of acetic acid in the final hydrolyzate depends on the composition and content of the hemicellulose fraction in the raw material, as well as the harshness of the treatment method [7]. Most lignocellulose hydrolyzates contain acetic acid at concentrations between 1 and 10 g·L^−1^, depending on the feedstock. Dilute-acid spruce hydrolyzates typically contain around 5 g·L^−1^ acetic acid [8], while hydrolyzates prepared from hardwood, sugar cane bagasse, corn stover, and wheat straw, generally contain more acetic acid, normally 9–10 g·L^−1^ [9,10,11,12]. Depending on the pH, total concentrations around 10 g·L^−1^ may cause significant inhibition to yeast growth and metabolism [13,14]. Much higher acetic acid concentrations, up to 17.5 g·L^−1^, have also been observed [15], and inhibition by acetic acid is a serious challenge to the utilization of many lignocellulosic raw materials. It is, therefore, important to find process conditions by which even substrates containing high acetic acid concentrations can be utilized.

Weak acids, such as acetic and benzoic acids, are traditionally used for food preservation, as they can reduce the presence of spoilage microorganisms [16]. The toxicity of acetic acid is strongly pH dependent, because the undissociated form of the acid is liposoluble and is able to diffuse through the cell membrane. Uptake of acetic acid has long been believed to occur through passive diffusion through the membrane [17], but recent studies propose that the Fps1p aquaglyceroporin channels can act as a facilitator for acetic acid uptake [18]. The lipid composition of the cell membrane can play an important role for the acetic acid tolerance of the yeast [19]. When undissociated acetic acid enters into the cytosol, where the pH is near neutral, it dissociates into a proton and an acetate ion. This lowers the cytoplasmic pH, which inhibits the activity of some enzymes [20], and increases the requirement for free energy to transport protons out from the cell [21]. In addition, the intracellular acetate ion is also toxic for the yeast cells [18], and requires ATP-dependent export via ABC transporters [16].

Some of the inhibitory effects of toxic compounds produced during hydrolysis can be overcome by appropriate design of the fermentation process. One interesting way is to use a bioreactor containing a very high yeast cell density [22,23]. High yeast cell density cultivations have been proven useful, e.g., for fermentation of media with high levels of furfural [23]. A high cell concentration can be achieved by using, e.g., immobilization or cell retention by membranes [23,24,25]. An ethanol yield equal to the theoretical yield on fermentable sugars, 0.51 g·g^−1^, was accomplished during fermentation of spruce hydrolyzate by cell recirculation using a cross-flow membrane filter [26]. The yeast cell concentration was 26 g·L^−1^, and the cells did not grow at the tested conditions. Earlier studies also show that the yeast growth and the specific ethanol productivity decrease with increasing yeast concentration during continuous cultures with cell recirculation [26,27]. The ideal situation would be if the fermentation conditions could be maintained such that a cell density providing maximum ethanol productivity and yield could be obtained, and that also allowed for a slow yeast growth only enough to compensate for dying cells.

Acetic acid and other weak acids present in hydrolyzate reduce the yeast growth rate but may also positively influence the specific ethanol productivity. The presence of acetic acid has been reported to be beneficial for improving the ethanol production, as long as the acetic acid concentration does not exceed the lethal concentration and a high growth rate is not required [26].

In the present study, anaerobic continuous cultivations were performed at both high and very high yeast cell densities by using a membrane bioreactor (MBR) with a cross-flow membrane. Continuous cultivations at acetic acid concentrations ranging from 2.5 to 20.0 g·L^−1^ were conducted at a rapid dilution rate of 0.5 h^−1^ and at pH 5.0. The goal of the study was to investigate the effects of acetic acid on ethanol production at high yeast cell densities. The aim was also to evaluate the use of a cross-flow membrane unit to retain the yeast cells and to achieve high volumetric ethanol productivity when using substrates containing high acetic acid concentrations.

## 2. Results and Discussion

### 2.1. Yeast Cell Dry Weight during Continuous Cultivations

The yeast cells in the MBR are exposed to very different conditions compared to the environment in a traditional continuous cultivation. By retaining the cells in the MBR the dilution rate is no longer limited by the growth rate of the yeast. In addition, the cell retention makes the process less sensitive to transient periods when cells cannot grow due to, high inhibitor concentration or pH fluctuations in the bioreactor.

The MBR system used in this study for retaining the cells had a stable and nonfouling operation which made it possible to run the cultivations at a dilution rate as high as 0.5 h^−1^ even at very high cell densities of up to approximately 200 g·L^−1^. By using very high yeast concentrations the sugars in the substrate medium can be fermented very rapidly. For example, sugarcane juice and molasses could be fermented to ethanol levels of 8%–11% (v/v) within 6–11 h by using high yeast concentrations of 10%–17% w/v (wet basis) [28].

The dilution rate could be kept at 0.5 h^−1^ during the entire study. After every cultivation the membrane module was cleaned with both hydrogen peroxide and sodium hypochlorite to maintain a high membrane permeability. Several studies have reported problems with the membrane modules when retaining the cells even at low dilution rates. For example, Palmquist *et al.* [26] operated at a maximum dilution of 0.1 h^−1^ with a cell density of 26 g·L^−1^, due to the limited capacity of the cross-flow membrane. Melzoch *et al.* [27] also reported low permeability of the employed membrane, with the consequence that cultivations could only be made at low biomass concentrations at dilution rates ranging from 0.03 to 0.2 h^−1^.

**Figure 1:**
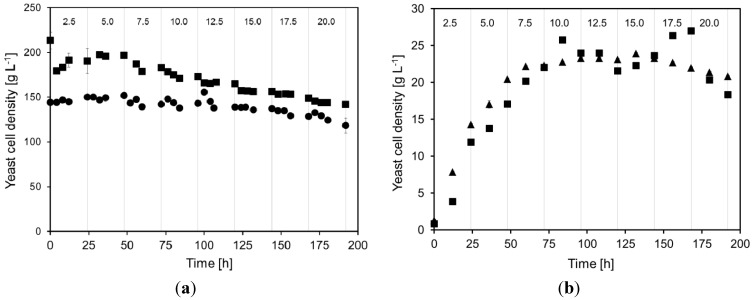
Yeast cells density in the MBR during two separate cultivations A (■) and B (▲), at very high (**a**) and high (**b**) yeast concentration. The total acetic acid concentration in the feed medium was changed every 24 h (vertical lines). The numbers at the top of the figure denote the approximate acetic acid concentration in the medium (in g·L^−1^) during each such 24 h period. The error bars in the graphs denote the maximum and minimum results for duplicate dry weight measurements.

The cell dry weight concentrations during duplicate continuous cultivations are shown in Figure 1. The maximum yeast cell density was 213 g·L^−1^ for cultivation A and 152 g·L^−1^ for cultivation B. However, both these cultivations at very high yeast density showed a falling cell density when the total acetic acid concentration in the feed medium was increased from 2.5 to 20.0 g·L^−1^. In cultivation A, the cell density decreased by approximately 30%, whereas in cultivation B it fell by approximately 20% during the 196 h long cultivation. Part of the reduced cell density in the cultivation was due to the biomass sampling from the cultivation, but not all. It is probable that the yeast cells either decreased in size or started to die due to the acetic acid stress or to insufficient supply of sugar or nutrients. A non-growing yeast is not necessarily negative for ethanol production, since biomass formation can be regarded as a by-product in the process. By retaining the cells, e.g., in a MBR, the process profits from lower biomass yield. In most cases, this means that a larger part of the raw material can be channeled to ethanol production, which is economically beneficial for the process [4].

In the cultivations at lower yeast concentration, the yeast did grow when the medium contained up to 10 g·L^−1^ acetic acid. The average specific growth rates in the two cultivations performed at high yeast density for each 24 h period at the acetic acid levels 2.5, 5.0, 7.5, and 10.0 g·L^−1^ were 0.48 ± 0.08, 0.015 ± 0.004, 0.007 ± 0.004, and 0.003 ± 0.001 h^−1^, respectively. After the initial rapid growth rate at 2.5 g·L^−1^ acetic acid it can clearly be seen that at each step increase of 2.5 g·L^−1^ acetic acid in the inlet medium, the average specific growth rate decreased by more than 50% during the following 24 h period. This can also be explained by the start-up procedure for the cultures at lower cell density, since the cells would grow until there is a balance between substrate addition, cell growth and maintenance metabolism, and the amount of cells removed by sampling and death [29].

### 2.2. Continuous Alcohol Fermentation

**Figure 2:**
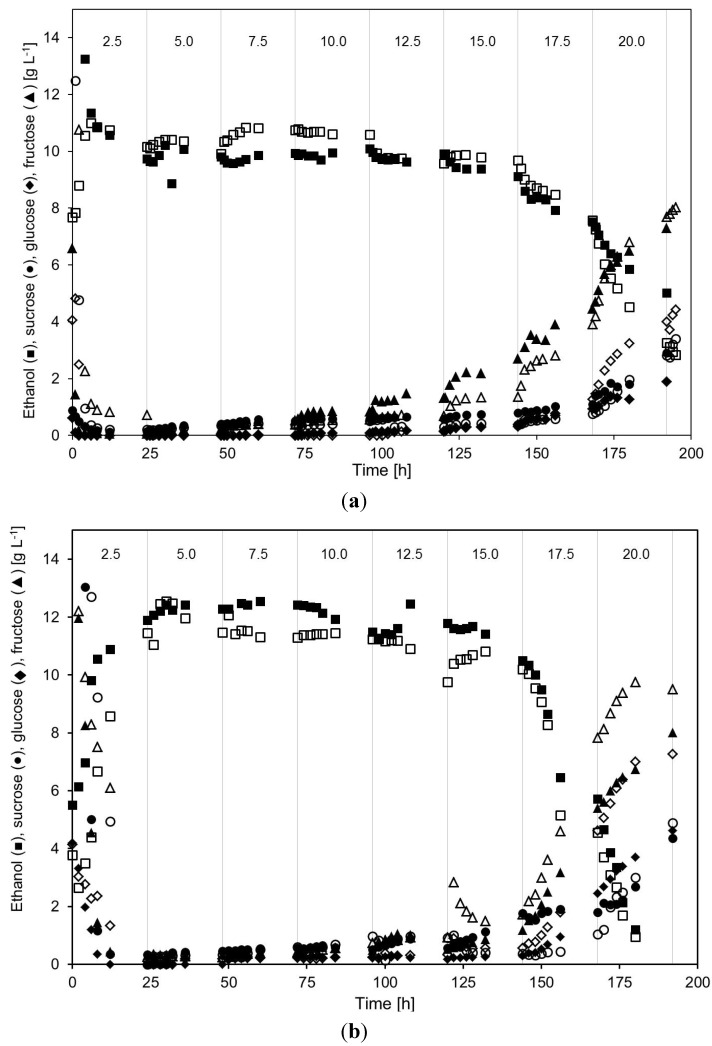
Concentration of ethanol, sucrose, glucose and fructose in the outflow from the MBR, during two separate cultivations (closed) and (open symbols) at very high (**a**) and high (**b**) yeast concentration. The total acetic acid concentration in the feed medium was changed every 24 h (vertical lines). The numbers at the top of the figures denote the approximate total acetic acid concentration (in g·L^−1^) during each such 24 h period.

**Figure 3:**
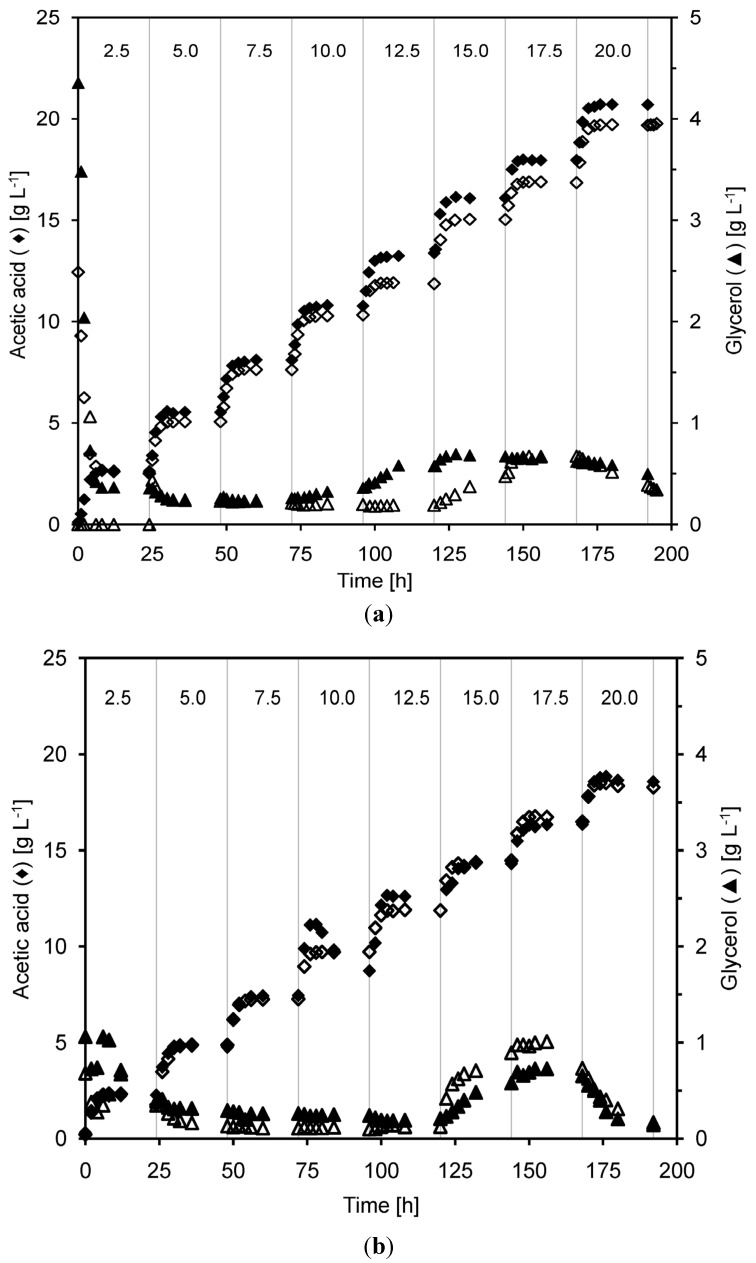
Concentration of glycerol and acetic acid in the outflow from the MBR, during two separate cultivations (closed) and (open symbols) at very high (**a**) and high (**b**) yeast concentration. The total acetic acid concentration in the feed medium was changed every 24 h (vertical lines). The numbers at the top of the figure denote the approximate total acetic acid concentration (in g·L^−1^) during each such 24 h period.

The ethanol fermentation profile of *S. cerevisiae* was compared under different acetic acid concentration. Measured values of fermentation products, sugars and acetic acid concentrations during the cultivations are presented in Figure 2 and Figure 3.

No differences could be seen in the acetic acid tolerance of the yeast, in terms of ethanol production, when cultivations were performed at either very high or high yeast concentrations. In both cases, the yeast was able to withstand acetic acid concentrations up to 15–16 g·L^−1^ without any large negative effects on the ethanol production, which is much higher than when no recirculation is done [14]. The ethanol concentration was, nevertheless, higher in the cultivations performed at a lower yeast cell concentration. At very high yeast concentration, the ethanol concentration remained around 10 g·L^−1^, whereas cultivations at the lower yeast concentration contained around 11–12 g·L^−1^ ethanol. It is unclear why the ethanol concentration was higher in the cultivations performed at lower yeast concentration. Ethanol is a primary product and, therefore, its production is linked to the growth of yeast cells. For non-growing yeast, the ethanol production has been reported to be at least 30 times slower [30]. However, according to classical theory on maintenance metabolism, the ethanol yield should be higher the lower the growth rate, since a larger part of the consumed substrate would be used for maintenance energy rather than for biosynthesis [31]. In the cultivations performed at high yeast concentration, in which the cells grew very slowly if at all yet still consumed most of the available sugar, the ethanol yield and concentration should therefore have been higher than in the cultivations at lower yeast concentration.

Even though growing yeast generally produce ethanol faster, the cultivations in this investigation illustrate that almost complete sugar fermentation with high ethanol yield could be achieved at both very high and high yeast cell concentrations even when the cells were not growing.

When the acetic acid concentration was increased above 15–16 g·L^−1^, with corresponds to 5.5–5.8 g·L^−1^ of undissociated acetic acid at pH 5.0, the ethanol concentration in the permeate decreased steadily, and even more severely at the highest acetic acid concentration. In the cultivations performed at lower yeast concentrations the ethanol decrease at the two highest acetic acid concentrations was much more severe compared with the cultivations containing high yeast concentrations.

The glycerol concentration in the filter permeate remained below 0.5 g·L^−1^ throughout more than half of the cultivation except for the initial phase, both at very high and high yeast density (Figure 3). An increased level of glycerol was measured when the level of acetic acid was increased to 15 g·L^−1^. At the highest acetic acid concentration the glycerol concentration decreased, probably because the yeast failed to continue fermenting the media. The increased glycerol production at 15 g·L^−1^ acetic acid concentration probably occurs due to an increased stress when the undissociated acetic acid concentration passes 5 g·L^−1^. It is known that yeast produce glycerol to counterbalance low water availability in hyperosmotic environments [32]. Lawrence *et al.* [33] observed that high concentrations of citric acid do not induce osmotic stress, but the presence of 150 mM and 200 mM citric acid instead induce a general stress response and also increased glycerol biosynthesis in *Saccharomyces cerevisiae*. This effect may be valid also under the high acetic acid concentrations used in this study.

Numerous investigations have been made to elucidate which toxic effects acetic acid has on *Saccharomyces cerevisiae*. When acetic acid enters the cell it reduces the intracellular pH, which can negatively affect several important cellular processes, such as the activity of glycolytic enzymes and NADH dehydrogenase [20,34]. Acetic acid may also trigger programmed cell death [35].

### 2.3. Product Yields and Ethanol Productivity

At very high yeast concentrations, the addition of acetic acid did not have any clear effect on the ethanol yield except at the two highest acetic acid concentrations (Table 1). In cultivations at the lower yeast concentration the ethanol yield increased from 0.43 to 0.51 g·g^−1^ (A) and from 0.35 to 0.48 g·g^−1^(B) when the acetic acid concentration was raised from 2.3 to 12.6 g·L^−1^ (A) and from 2.3 to 11.9 g·L^−1^ (B), respectively (Table 2). However, when there was more than 14.5 g·L^−1^ acetic acid in the medium the ethanol yield decreased, and at the highest acetic acid concentrations the yield was only 0.1–0.3 g·g^−1^. These findings are in accordance with Taherzadeh *et al.* [14], who report an increase in ethanol yield from 0.39 to 0.45 g·g^−1^ in batch cultivations when the amount of undissociated acetic acid was increased from 0 to 3.3 g·L^−1^ (corresponding to a total acetic acid concentration of 0 to 9 g·L^−1^) [14].

The opposite tendency was seen in the glycerol yield, which instead decreased as the acetic acid content was stepwise increased in the cultivation to about 11.9 g·L^−1^. Above this level, the glycerol yield increased again (Figure 2, Table 1 and Table 2). In batch cultivations containing acetic acid the glycerol yield has been shown to be 0.05 g·g^−1^ at 3 g·L^−1^ undissociated acetic acid which is much higher than in this study where it was around 0.01 g·g^−1^ [14]. The low glycerol yields observed here are most likely due to the very low growth rate in the high cell density cultures, since glycerol is produced to reoxidize excess NADH produced in biosynthesis [36]. Taherzadeh *et al.* also reported that the glycerol yield decreased from 0.09 to 0.05 g·g^−1^ when the total acetic acid concentration was increased from 0 to 9 g·L^−1^, and explained this by reduced biomass yields [14]. The ethanol and glycerol yields can also differ extensively depending on which yeast strain is used in acetic acid containing medium. For example, Gilbert *et al.* reported ethanol yields ranging from 0.41 to 0.32 g·g^−1^ and glycerol yields from 0.061 to 0.034 g·g^−1^ when several different yeast strains were investigated [37].

**Table 1:** Summary of product yields and ethanol productivity during continuous fermentations at very high yeast concentrations.

Acetic Acid conc. (g·L^−1^)		Ethanol (g·L^−1^)	Sugar ^a^ (g·L^−1^)	Glycerol (g·L^−1^)	Biomass (g·L^−1^)	Y_p/s_ (g·g^−1^)	Y_gly/s_ (g·g^−1^)	r_p_ (g·L^−1^·h^−1^)	q_p_ (g·g^−1^·h^−1^)	η (%)
Total	Undissociated at pH 5	Cultivation A								
2.6	0.9	9.73	0.20	0.35	190.6	0.39	0.016	4.87	0.026	99.2
5.5	2.0	9.81	0.62	0.26	196.6	0.40	0.012	4.91	0.025	97.6
8.1	3.0	9.94	1.04	0.26	183.2	0.42	0.012	4.97	0.027	95.8
10.8	3.9	10.08	1.49	0.36	173.2	0.43	0.017	5.04	0.029	94.0
13.4	4.9	9.91	2.04	0.57	165.2	0.44	0.028	4.96	0.030	91.8
16.1	5.9	9.12	3.88	0.67	156.5	0.43	0.033	4.56	0.029	84.6
18.0	6.6	7.53	6.49	0.62	149.0	0.41	0.034	3.77	0.025	73.7
20.7	7.6	5.01	12.09	0.50	141.6	0.38	0.032	2.51	0.018	52.2
Total	Undissociated at pH 5	Cultivation B								
2.5	0.9	10.15	0.94	0.45	150.0	0.43	0.020	5.08	0.034	96.2
5.1	1.9	9.91	0.55	0.23	151.9	0.40	0.010	4.96	0.033	97.8
7.6	2.8	10.74	0.76	0.21	142.3	0.44	0.010	5.37	0.038	97.0
10.3	3.8	10.58	1.08	0.20	143.3	0.43	0.009	5.29	0.037	95.7
11.9	4.3	9.76	1.15	0.19	139.0	0.43	0.009	4.88	0.035	95.1
15.0	5.5	9.68	2.04	0.47	137.3	0.42	0.022	4.84	0.035	91.9
16.8	6.1	7.57	5.94	0.67	128.6	0.39	0.036	3.79	0.029	76.7
19.7	7.2	3.27	14.51	0.39	118.6	0.31	0.035	1.64	0.014	42.1

Notes: All results are based on values after adding feed medium with the indicated acetic acid concentration for 24 h. Concentrations of ethanol, sugar and glycerol are at the membrane outlet. ^a^ Sum of residual sucrose, fructose and glucose; Y_p/s_ = Ethanol yield on sugars consumed; Y_gly/s_ = Glycerol yield on consumed sugars; r_p_ = Volumetric ethanol productivity; q_p_ = Specific ethanol production; η = Sugar utilization.

The specific ethanol production rates were very low (0.014–0.38 g·g^−1^·h^−1^), which was to be expected due to the high cell densities. In for example batch cultivations with acetic acid specific ethanol productivity of for example 2.7 g·g^−1^·h^−1^ has been reported [14]. Nevertheless, as a consequence of the high dilution rate, fairly high volumetric ethanol productivity could be reached during the cultivation, even when the medium only contained 25 g·L^−1^ sucrose. The maximum volumetric ethanol productivities were 5.0–5.4 g·L^−1^·h^−1^ at 7–11 g·L^−1^ acetic acid in the very high cell density cultures, and 5.7–6.2 L^−1^·h^−1^ in the high cell density cultures. At an acetic acid level over 13–15 g·L^−1^ the volumetric productivity started to drop, probably because a toxic concentration of acetic acid was reached. When medium containing no acetic acid was supplied the volumetric productivity increased again.

At the lower acetic acid concentrations, the sucrose in the medium was almost completely consumed and there were only low amounts of glucose, fructose and sucrose left in the filter permeate (Figure 2). As the acetic acid concentration in the medium was increased to about 12–15 g·L^−1^ more unconsumed sugars became present in the permeate (Table 1 and Table 2).

**Table 2:** Product yields and ethanol productivity during continuous fermentations at high yeast concentrations.

Acetic Acid conc. (g·L^−1^)		Ethanol (g·L^−1^)	Sugar ^a^ (g·L^−1^)	Glycerol (g·L^−1^)	Biomass (g·L^−1^)	Y_p/s_ (g·g^−1^)	Y_gly/s_ (g·g^−1^)	r_p_ (g·L^−1^·h^−1^)	q_p_ (g·g^−1^·h^−1^)	η (%)
Total	Undissociated at pH 5	Cultivation A								
2.3	0.9	10.89	0.65	0.39	14.3	0.43	0.015	5.45	0.381	0.98
4.8	2.0	12.32	0.79	0.29	20.4	0.48	0.011	6.16	0.302	0.97
7.4	3.0	12.29	1.25	0.26	22.3	0.49	0.010	6.15	0.276	0.95
10.8	3.9	11.93	1.23	0.24	23.3	0.48	0.010	5.96	0.256	0.95
12.6	4.9	11.62	1.27	0.21	23.1	0.51	0.009	5.81	0.252	0.95
14.3	5.9	11.43	3.25	0.58	23.3	0.50	0.025	5.71	0.246	0.88
16.4	6.6	6.46	9.65	0.65	21.9	0.39	0.039	3.23	0.148	0.63
18.9	7.6	1.19	17.00	0.14	20.8	0.13	0.015	0.60	0.029	0.35
Total	Undissociated at pH 5	Cultivation B								
2.3	0.9	8.58	0.27	0.35	11.9	0.35	0.014	4.29	0.360	0.99
4.9	1.9	11.98	0.76	0.13	17.1	0.50	0.005	5.99	0.350	0.97
7.3	2.8	11.31	1.23	0.11	22.0	0.48	0.005	5.66	0.257	0.95
9.7	3.8	11.45	1.95	0.10	24.0	0.50	0.004	5.73	0.239	0.92
11.9	4.3	10.90	2.19	0.12	21.6	0.48	0.005	5.45	0.252	0.91
14.5	5.5	10.82	2.66	0.89	23.6	0.49	0.040	5.41	0.229	0.89
16.5	6.1	5.15	13.51	0.73	27.0	0.45	0.064	2.58	0.095	0.46
18.3	7.2	0.95	21.68	0.17	18.3	0.29	0.053	0.48	0.026	0.13

Notes: All results are based on values after adding feed medium with the indicated acetic acid concentration for 24 h. Concentrations of ethanol, sugar and glycerol are at the membrane outlet. ^a^ Sum of residual sucrose, fructose and glucose; Y_p/s_ = Ethanol yield on sugars consumed; Y_gly/s_ = Glycerol yield on consumed sugars; r_p_ = Volumetric ethanol productivity; q_p_ = Specific ethanol production; η = Sugar utilization.

### 2.4. Applicability of MBR for 2nd Generation Lignocellulosic Bioethanol Production

Our results indicate that using a MBR may be useful for fermentation of strongly inhibitory media obtained from lignocellulosic materials. The main obstacle in MBR systems is membrane fouling. Some ways to deal with this problem is to apply high cross-flow velocities and chemical or physical cleaning [38]. Consequently, it may be problematic to use MBR systems if the medium contain fibers or particles since they will accumulate inside the MBR.

One advantage of the MBR systems is that the final product stream is free from cells which makes the downstream processing easier. An earlier study also showed that high yeast density cultivations performed in a MBR can withstand very high furfural levels of up to 17 g·L^−1^ in the feed medium at a dilution rate of 0.5 h^−1^ [23]. Furfural is a fermentation inhibitor which can be detoxified *in situ* by the yeast [6]. By applying a high density cultivation in the MBR a high tolerance for convertible inhibitors and fast *in situ* detoxification can be achieved [23]. MBR systems may also be advantageous for all inhibitors, which can be bioconverted by the yeast. However, our study shows that even for the nonconvertible inhibitor acetate the inhibitor tolerance of the yeast in a MBR system is high.

## 3. Materials and Methods

A series of continuous cultivations were performed with *S. cerevisiae* using complex sucrose media containing different amounts of acetic acid. The cells were retained in the bioreactor by means of an external cross-flow membrane module. Cell-free permeate, containing fermentation products and unfermented sugars, was removed through the membrane. Except for sampling to measure the cell density inside the bioreactor, the yeast was retained inside the membrane bioreactor. The response of *S. cerevisiae* was investigated by measuring the concentrations of extracellular metabolites.

### 3.1. Microorganism and Medium

The yeast strain *Saccharomyces cerevisiae* CBS 8066, acquired from Centraalbureau voor Schimmelcultures (Delft, The Netherlands) was used throughout the investigation. The strain was maintained on YPD agar plates containing 10 g·L^−1^ yeast extract (Scharlau, Sentmenat, Spain), 20 g·L^−1^ soy peptone (Fluka, Steinheim, Germany), 20 g·L^−1^ d-glucose (Fisher Scientific, Leicestershire, UK), and 20 g·L^−1^ agar (Scharlau, Sentmenat, Spain), and stored refrigerated at 4 °C until use.

The medium used during cultivations contained 1 g·L^−1^ yeast extract, 2 g·L^−1^ (NH_4_)_2_SO_4_, 5 g·L^−1^ KH_2_PO_4_, 0.4 g·L^−1^ MgSO_4_·7H_2_O, and 0.1 mL·L^−1^ antifoam SE-15 (Sigma, St. Louis, MI, USA) to avoid foaming. Sucrose (from *Beta vulgaris*, Dan Sukker, Örtofta, Sweden) was used as carbon source and added to final concentrations of 25 or 100 g·L^−1^. Sterile glacial acetic acid (Scharlau, Sentmenat, Spain) was mixed with the medium after autoclaving to obtain total acetic acid concentrations ranging from 2.5–20 g·L^−1^. In order to avoid sugar degradation and unwanted chemical reactions, salt solution, antifoam, yeast extract, and sugar solution were autoclaved separately prior to mixing in a 5 L blue-cap flask. For inoculum cultures, medium with 100 g·L^−1^ sucrose was used. 200 mL of the medium was added in 500 mL cotton plugged E-flasks to which one yeast colony was added. After inoculation, the E-flasks were placed in a water bath at 30 °C and 130 rpm for 24 h.

### 3.2. Dry Cell Weight Determination

Samples for dry weight determination were removed directly from the continuous cultivation via the sampling port of the bioreactor. The tube containing yeast suspension was centrifuged (9600× *g*, 5 min), the supernatant was removed from the tube and the cell pellet was stored at −20 °C until analysis. Before transferring the cell biomass samples to preweighed glass tubes they were washed three times with Milli-Q water to remove salts and other soluble compounds. The samples were then placed in an oven at 105 °C for 24 h and weighed after temperature equilibration in a desiccator.

### 3.3. Sugar and Extracellular Metabolite Analysis

Liquid samples to determine sugar, inhibitor and extracellular metabolite concentrations in the MBR were taken from the permeate outflow from the cross flow membrane unit and stored at −20 °C until analysis. Quantification of the compounds was performed by HPLC (Waters 2695, Waters Corporation, Milford, MA, USA) using an Aminex HPX87-H column (Bio-Rad Laboratories, München, Germany) for extracellular metabolite and inhibitor concentrations, and an Aminex HPX87-P column (Bio-Rad Laboratories, München, Germany) for sugar concentrations. A refractive index detector (Waters 2410, Waters Corporation, Milford, MA, USA) was used to detect all components. The Aminex HPX87-H column was maintained at 60 °C and eluted with 5 mM H_2_SO_4_ at a flow rate of 0.6 mL·min^−1^, whereas the Aminex HPX87-P column was kept at 85 °C using MilliQ water at an eluent flow rate of 0.6 mL·min^−1^.

### 3.4. Cross-Flow Membrane Unit

In the MBR, an external tubular cross-flow membrane filtration unit (dizzer^®^ LAB 1.5 MB 0.1 inge Gmbh, Greifenberg, Germany) with a surface area of 0.1 m^2^ was used to retain the cells in the bioreactor and at the same time remove fermented medium from the cultivation. The filter was made of polyethersulfone with a pore size of approximately 0.02 μm. Prior to each run, the membrane unit was first cleaned with 500 mg·L^−1^ H_2_O_2_, disinfected with sodium hypochlorite solution containing 100 mg·L^−1^ active chlorine, and rinsed with sterile water to remove the chlorine.

### 3.5. MBR Cultivations

**Figure 4:**
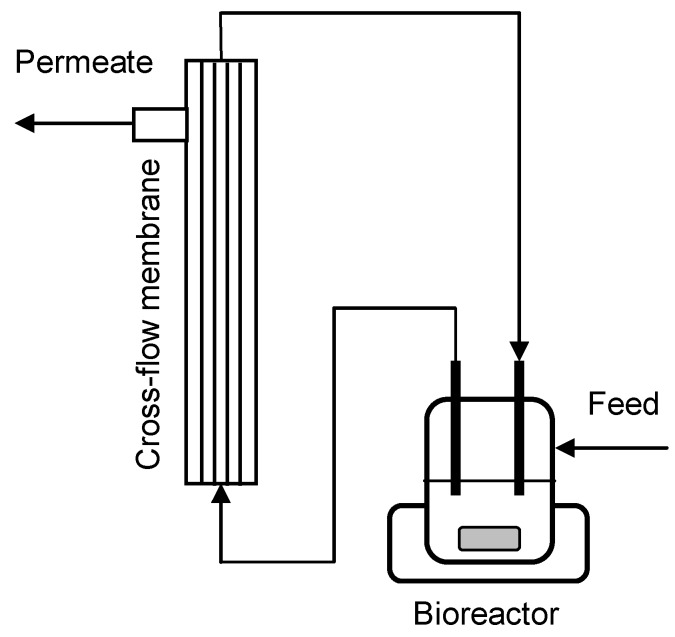
Schematic design of the MBR. The cross-flow membrane is externally coupled to the bioreactor, which is mixed with a magnetic impeller and heated with a water containing metal collar.

The schematic design of the MBR (cross flow membrane unit) is presented in Figure 4. The acetic acid tolerance of the yeast cells was screened by increasing the total acetic acid concentration in the medium reservoir stepwise by 2.5 g·L^−1^ each 24 h, to a total acetic acid concentration of 20 g·L^−1^. Eight different acetic acid concentrations were tested namely 2.5, 5.0, 7.5, 10.0, 12.5, 15.0, 17.5, and 20.0 g·L^−1^, corresponding to 0.9–7.3 g·L^−1^ undissociated acetic acid at pH 5. For cultivations performed at “very high” yeast concentrations, the MBR was started by transferring 400 mL yeast inoculum culture into the bioreactor (200 mL SARA fermentor, Belach Bioteknik, Stockholm, Sweden). The yeast was propagated aerobically for 72 h by feeding medium with 100 g·L^−1^ sucrose at a dilution rate of 0.5 h^−1^. The system was set to anaerobic conditions by sparging with pure nitrogen gas for 15 min before changing the medium to acetic acid containing medium with 25 g·L^−1^ sucrose. When performing cultivations at high yeast density only 150 mL of yeast inoculum and 150 mL 0.9% NaCl was added to the bioreactor, and the yeast was not propagated before addition of acetic acid containing medium with 25 g·L^−1^ sucrose, which was subsequently used throughout the cultivation.

During cultivation, the total liquid volume inside the bioreactor and the membrane unit was kept at 0.3 L by removing consumed broth through the cross-flow membrane unit. In the cross-flow membrane unit the fermentation broth was continuously circulated with a velocity of ~0.5 L·min^−1^, corresponding to a linear velocity of 0.08 m·s^−1^, with the help of a peristaltic pump, to reduce membrane fouling and provide a sufficiently high filtration rate. The total liquid volume inside the MBR was kept constant by connecting a level control inside the bioreactor to a harvesting pump, which removed culture broth through the cross-flow membrane. New medium was continuously fed into the bioreactor at a dilution rate of 0.5 h^−1^, based on the total volume of liquid in the reactor and in the membrane unit. The control unit of a Biostat A. bioreactor (Braun Biotech, Melsungen, Germany) was used to maintain the pH at 5.0 by addition of 2 M NaOH and the temperature at 30 °C. The stirring rate inside the SARA fermentor was kept at 400 rpm and 200 mL·min^−1^ of either air (aerobic) or nitrogen (anaerobic) was continuously sparged through the medium. The oxygen content in the nitrogen gas (AGA Sweden, Göteborg, Sweden) was less than 5 ppm.

## 4. Conclusions

(1) In relation to ethanol production, no major differences could be seen in the acetic acid tolerance of the yeast cultivated either at very high or high yeast concentrations. In the employed MBR system, both very high and high cell density yeast cultivations were able to withstand total acetic acid concentrations of up to 15–16 g·L^−1^ at pH 5.0 without any major impact on the ethanol production. However, at even higher acetic acid concentrations, the ethanol production started to decrease.
(2) The cross-flow membrane showed a very stable permeability even at high yeast cell densities and the rapid dilution rate of 0.5 h^−1^, corresponding to a permeate outflow of 1.5 L·m^−2^·h^−1^ during 196 h. No particular cleaning of the module was required during the cultivation.
(3) Because the cultivations could be performed at a high dilution rate of 0.5 h^−1^ a high volumetric ethanol productivity of 5.04–6.16 g·L^−1^·h^−1^ could be reached at very high yeast concentrations even when only 25 g·L^−1^ sucrose medium was fed to the MBR. This indicates that the MBR system can be used to reach high ethanol productivity, which is crucial for successful bioethanol production from lignocelluloses.
(4) Very high yeast cell densities, up to 200 g dry weight L^−1^, could be maintained in the MBR.
